# W. J. M. Gordon

**Published:** 1875-06

**Authors:** 


*W. J. M. Gordon.*

No carpenter can do good work with poor tools. If this is true of inanimate objects, wood and iron, how much more true when applied to that wonderful piece of mechanism, the human body. The carelessness with which some druggists select their stock has done immense harm to the profession, and it is a duty all owe the noble brotherhood to praise the good, when we find it, as much as we blame the bad. A short sketch of the gentleman whose name heads this article will not be out of place in these pages.

Mr. Gordon was born in Maryland, and studied chemistry, under Prof. Wm. E. Aiken, of the Maryland University. He removed to Cincinnati in 1848, where he began business as a retail apothecary, and was remarkably successful. Not content with a mere pecuniary success, he soon started a laboratory connected with his store, and manufactured all the new chemical and pharmaceutical preparations as soon as they were brought to notice in the foreign or American journals. Never satisfied until he had the *best* and *purest* preparation possible, he was soon obliged to discontinue his retail store, and devote himself entirely to the manufacture of chemicals. He now stands at the head of his profession—his name on a package, a gurantee of its genuineness. His glycerine has taken the premium wherever it has been exhibitee, and he is the pioneer in the West in the manufacture of fluid extracts. His faciiities for making these are so great that he can not only compete with Eastern houses, but even suppiies some of them with his preparations. To give some idea of his vast establishment, we from the *Lancet and Observer:* "Mr. Gordon pays over $30,000 a year to one house in this city, for refuse material used in the manufacture of glycerine, which formerly went out into she city sewers." Mr. Gordon manufactured nitro-glycerine before it was used as an explosive. It was then called "glonine," and was used by the homeopaths as a remedy ln headache. Our limits do not allow us to notice many other preparations equally as interesting; but we take great pleasure in referring our readers to Mr. Gordon's advertisement, and can assure them that his goods are exactly what they are represented to be. As we consider it as much the duty of a medical journal to direct the attentiou of the profession to pure drugs as to guide them in their use when found, we shall from time to time notice all the houses whose goods we can recommend, and will not spare our connemnation of those who flood the stores with impure preparations. L.

